# Glycogen Synthase Kinase-3 regulates IGFBP-1 gene transcription through the Thymine-rich Insulin Response Element

**DOI:** 10.1186/1471-2199-5-15

**Published:** 2004-09-06

**Authors:** David Finlay, Satish Patel, Lorna M Dickson, Natalia Shpiro, Rodolfo Marquez, Chris J Rhodes, Calum Sutherland

**Affiliations:** 1 Department of Pathology and Neurosciences, University of Dundee, Ninewells Medical School and Hospital, Dundee, DD1 9SY United Kingdom; 2 Pacific Northwest Research Institute, 720 Broadway, Seattle, WA 98122, USA; 3 Division of Biological Chemistry, School of Life Sciences, University of Dundee, DD1 4EH, United Kingdom; 4 Ontario Cancer Institute, University of Toronto, 610 University Avenue, Toronto, Ontario M5G 2M9 Canada

**Keywords:** GSK-3, Insulin, IGFBP-1 gene transcription, TIRE, CHIR99021

## Abstract

**Background:**

Hepatic expression of several gene products involved in glucose metabolism, including phosphoenolpyruvate carboxykinase (PEPCK), glucose-6-phosphatase (G6Pase) and insulin-like growth factor binding protein-1 (IGFBP-1), is rapidly and completely inhibited by insulin. This inhibition is mediated through the regulation of a DNA element present in each of these gene promoters, that we call the Thymine-rich Insulin Response Element (TIRE). The insulin signalling pathway that results in the inhibition of these gene promoters requires the activation of phosphatidylinositol 3-kinase (PI 3-kinase). However, the molecules that connect PI 3-kinase to these gene promoters are not yet fully defined. Glycogen Synthase Kinase 3 (GSK-3) is inhibited following activation of PI 3-kinase. We have shown previously that inhibitors of GSK-3 reduce the activity of two TIRE-containing gene promoters (PEPCK and G6Pase), whose products are required for gluconeogenesis.

**Results:**

In this report we demonstrate that in H4IIE-C3 cells, four distinct classes of GSK-3 inhibitor mimic the effect of insulin on a third TIRE-containing gene, IGFBP-1. We identify the TIRE as the minimum requirement for inhibition by these agents, and demonstrate that the target of GSK-3 is unlikely to be the postulated TIRE-binding protein FOXO-1. Importantly, overexpression of GSK-3 in cells reduces the insulin regulation of TIRE activity as well as endogenous IGFBP-1 expression.

**Conclusions:**

These results implicate GSK-3 as an intermediate in the pathway from the insulin receptor to the TIRE. Indeed, this is the first demonstration of an absolute requirement for GSK-3 inhibition in insulin regulation of gene transcription. These data support the potential use of GSK-3 inhibitors in the treatment of insulin resistant states such as Type 2 diabetes mellitus, but suggest that it will be important to identify all TIRE-containing genes to assess potential side effects of these agents.

## Background

Insulin-like growth factors (IGF-I and II) have a broad range of biological activities that include the stimulation of mitogenesis and differentiation, and insulin-like effects on glucose uptake and lipogenesis [1]. These activities are modulated by a family of six binding proteins, termed the IGF-binding proteins (IGFBPs 1–6) that bind IGF-I and IGF-II with high affinity (for review see [2]). IGFBP-1 binds and inhibits the activity of IGF-I and IGF-II in plasma, by regulating their bioavailability [3]. Administration of excess IGFBP-1, or overexpression of IGFBP-1 in transgenic mice, leads to glucose intolerance and hyperinsulinaemia [4,5]. Meanwhile, IGFBP-1 expression can be dynamically regulated by nutritional status, increasing during fasting, malnutrition and diabetes but decreasing upon re-feeding or insulin treatment [6-8]. Hepatic IGFBP-1 gene transcription is rapidly and completely inhibited by insulin [9,10], however, the signalling pathway(s) that mediates this effect is less well defined. Insulin induces multiple intracellular signalling pathways in liver. Stimulation of the small G-protein Ras leads to activation of a protein kinase cascade consisting of Raf-1, MAP kinase kinase-1, p42/p44 MAP kinases and p90Rsk, while the activation of phosphoinositide (PI) 3-kinase promotes the generation of 3-phosphoinositides that induce the activity of protein kinases such as 3-phosphoinositide dependent kinase (PDK1) and protein kinase B (PKB) [11,12]. PKB subsequently phosphorylates glycogen synthase kinase -3 (GSK-3) at an N-terminal serine residue (Ser-21 on GSK-3α and Ser-9 on GSK-3β) rendering it inactive [13,14]. This PKB-mediated inhibition of GSK-3 contributes to insulin activation of glycogen and protein synthesis [14,15].

Studies using inhibitors of PI 3-kinase have demonstrated a requirement for this enzyme in insulin regulation of IGFBP-1 [16]. Indeed, overexpression of an active mutant of PKB mimics the effects of insulin on the IGFBP-1 promoter [16]. This effect, at least in part, is mediated through the inhibition of a Thymine-rich Insulin Response Element (TIRE) that lies between residues -120 and -96 relative to the transcription start site of the human gene promoter. Phosphoenolpyruvate carboxykinase (PEPCK) and Glucose-6-Phosphatase (G6Pase), rate-controlling enzymes of hepatic gluconeogenesis, possess a related regulatory element within their gene promoters [17]. Interestingly, members of the FOX(O) family of transcription factors (FKHR/FKHR-L1/AFX) have been linked to the regulation of the TIRE's found in these promoters [18,19]. The expression of all of these genes, as well as the regulation of FOX(O), is inhibited by insulin through a PI 3-kinase-dependent mechanism [20-24], suggesting that a common signalling pathway is utilised by insulin to regulate these related TIREs. However, insulin regulation of IGFBP-1 but not G6Pase or PEPCK gene expression is sensitive to an inhibitor of the mammalian Target of Rapamycin (mTOR) [10,25]. In addition, agents that strongly induce the MAPK pathway (e.g. phorbol esters) [26], as well as the protein phosphatase inhibitor okadaic acid [27], reduce the sensitivity of the IGFBP-1, but not the G6Pase and PEPCK promoters to insulin. Therefore, aspects of the signalling networks used by insulin to repress each of these TIRE containing promoters appear distinct. Recently, we observed that GSK-3 activity was required for both PEPCK and G6Pase promoter activity [28]. Selective inhibitors of GSK-3 reduce PEPCK and G6Pase gene transcription without requiring the activation of PKB. Indeed, the inhibition of GSK-3 may explain some of the effects of PKB overexpression on PEPCK and G6Pase gene expression. However, it was not clear why inhibition of GSK-3 should repress these promoters, whether inhibition of GSK-3 was actually required for insulin regulation of the genes, and whether the effect of GSK-3 inhibition was mediated through the TIRE.

In the present study, we have examined the role of GSK-3 in the regulation of a third TIRE-containing gene promoter, namely IGFBP-1. We demonstrate that four different classes of inhibitors of GSK-3 can mimic the action of insulin and reduce IGFBP-1 gene expression. Furthermore, we find that inhibition of GSK-3 reduces the activity of a heterologous promoter containing the IGFBP-1 TIRE, and propose that this mechanism underlies the repression of all three promoters by inhibitors of GSK-3. Finally, we demonstrate for the first time a requirement for inhibition of GSK-3 in the insulin regulation of the TIRE, and hence IGFBP-1 expression.

## Results

### Lithium ions reduce IGFBP-1 gene expression in H4IIE cells

**Figure 1:**
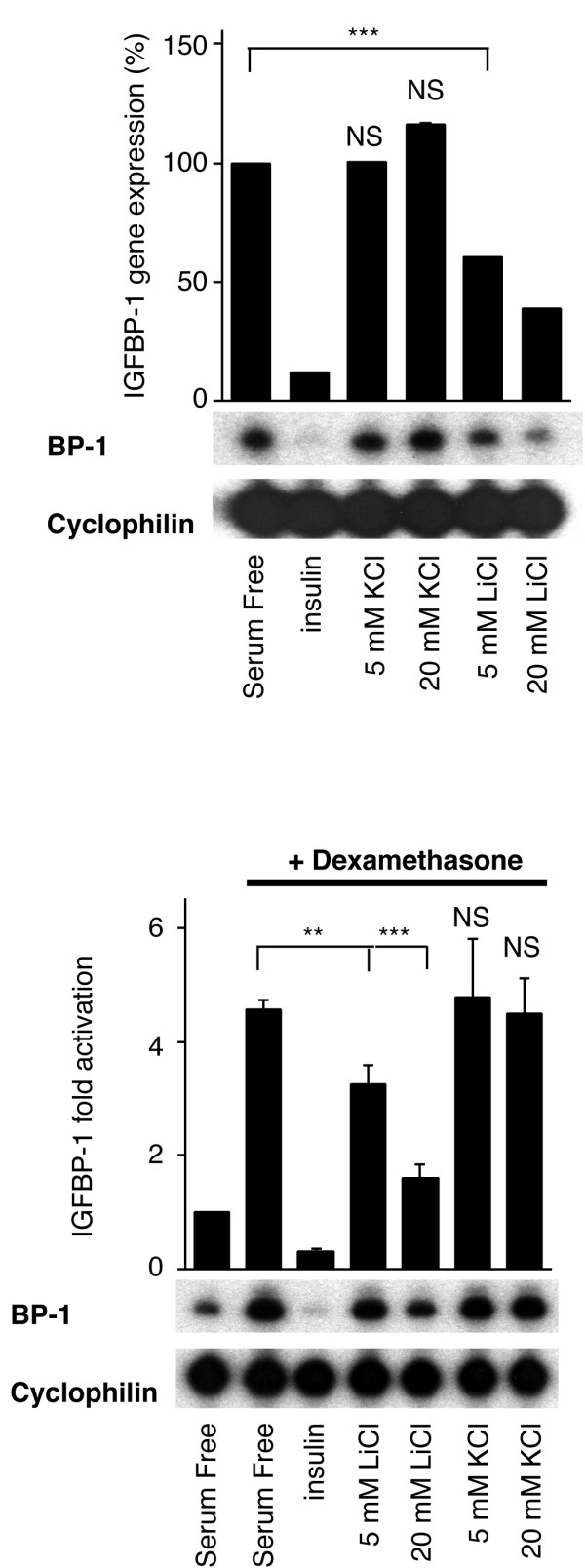
*Lithium ions reduce IGFBP-1 gene expression*. H4IIE cells were starved overnight prior to a 3 h incubation with insulin,10 nM; lithium chloride or potassium chloride at the concentrations indicated with or without dexamethasone, 500 nM; (A-B). Total cellular RNA was isolated and an RNase protection assay was performed as described in material and methods. Results are presented as percentage gene expression (A) or fold induction (B) relative to control and are means ± standard error of two experiments performed in duplicate (upper panels). Representative experiments (lower panels) are also shown. ***, p < 0.001, **, p < 0.01 and NS, not significant

Treatment of H4IIE cells with insulin completely inhibits both basal and glucocorticoid-induced IGFBP-1 gene expression. Lithium chloride, an inhibitor of GSK-3 *in vivo*, reduces both basal and glucocorticoid-induced IGFBP-1 gene expression (Fig 1). The effect of 20 mM lithium is not as complete as observed with insulin, resulting in only a 60–70% reduction of IGFBP-1 gene expression. However, treatment of H4IIE cells with the same concentration of potassium chloride has no effect on IGFBP-1 expression. The cyclophilin mRNA levels remain unchanged throughout these experiments. This highlights a role of a target of lithium ions in the specific regulation of IGFBP-1 gene expression.

### More selective inhibitors of GSK-3 also reduce IGFBP-1 gene expression

**Figure 2:**
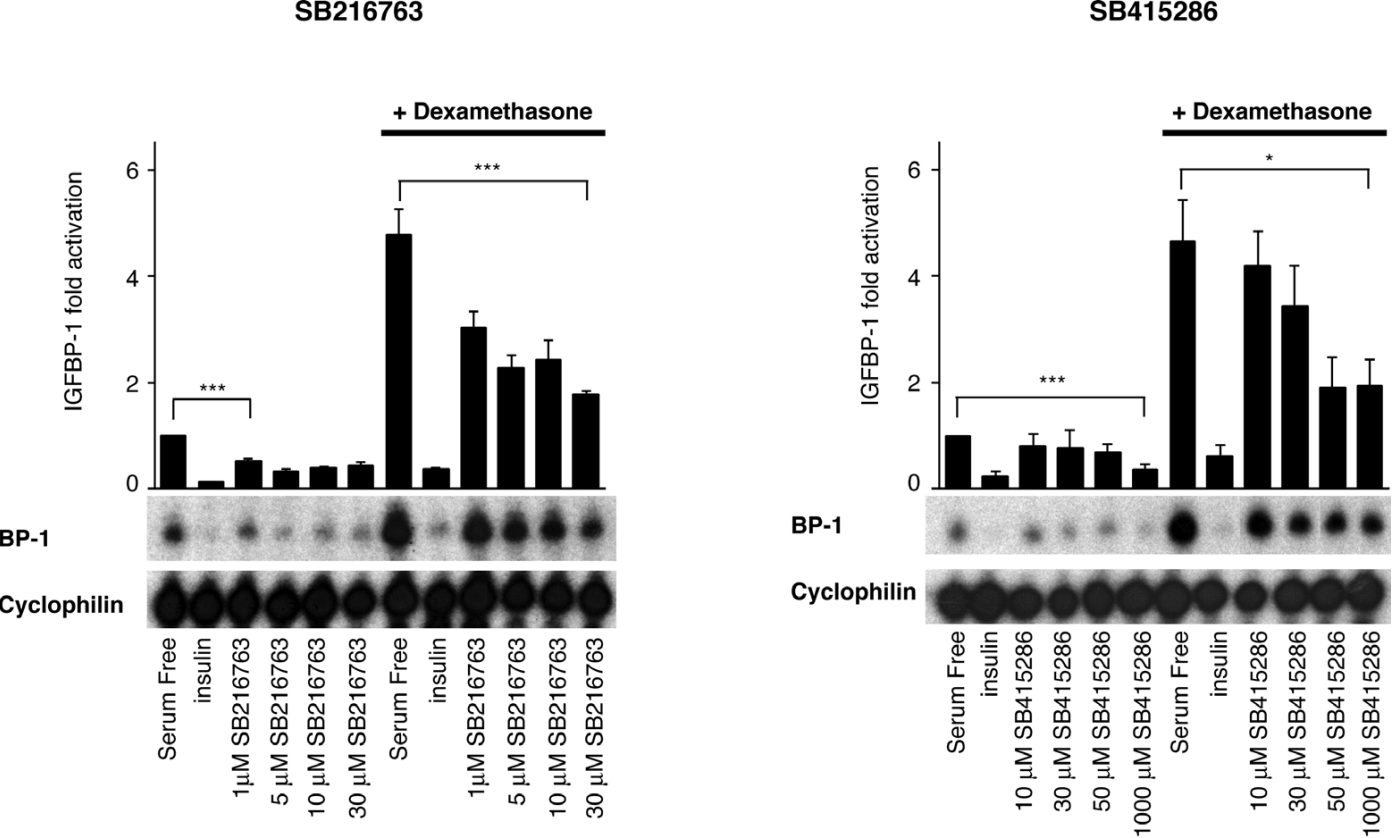
*SB216763 and SB415286 reduce IGFBP-1 gene expression*. H4IIE cells were starved overnight prior to a 3 h incubation with insulin, 10 nM ; dexamethasone, 500 nM; plus or minus SB216763 (A) or SB415286 (B) at the concentrations shown. Total cellular RNA was isolated and an RNase protection assay was performed, as described in material and methods. Results are presented as fold induction relative to control (serum free) and are means ± standard error of two experiments performed in duplicate (upper panels). Representative experiments are also shown (lower panels). ***, p < 0.001 and * p < 0.05

**Figure 3:**
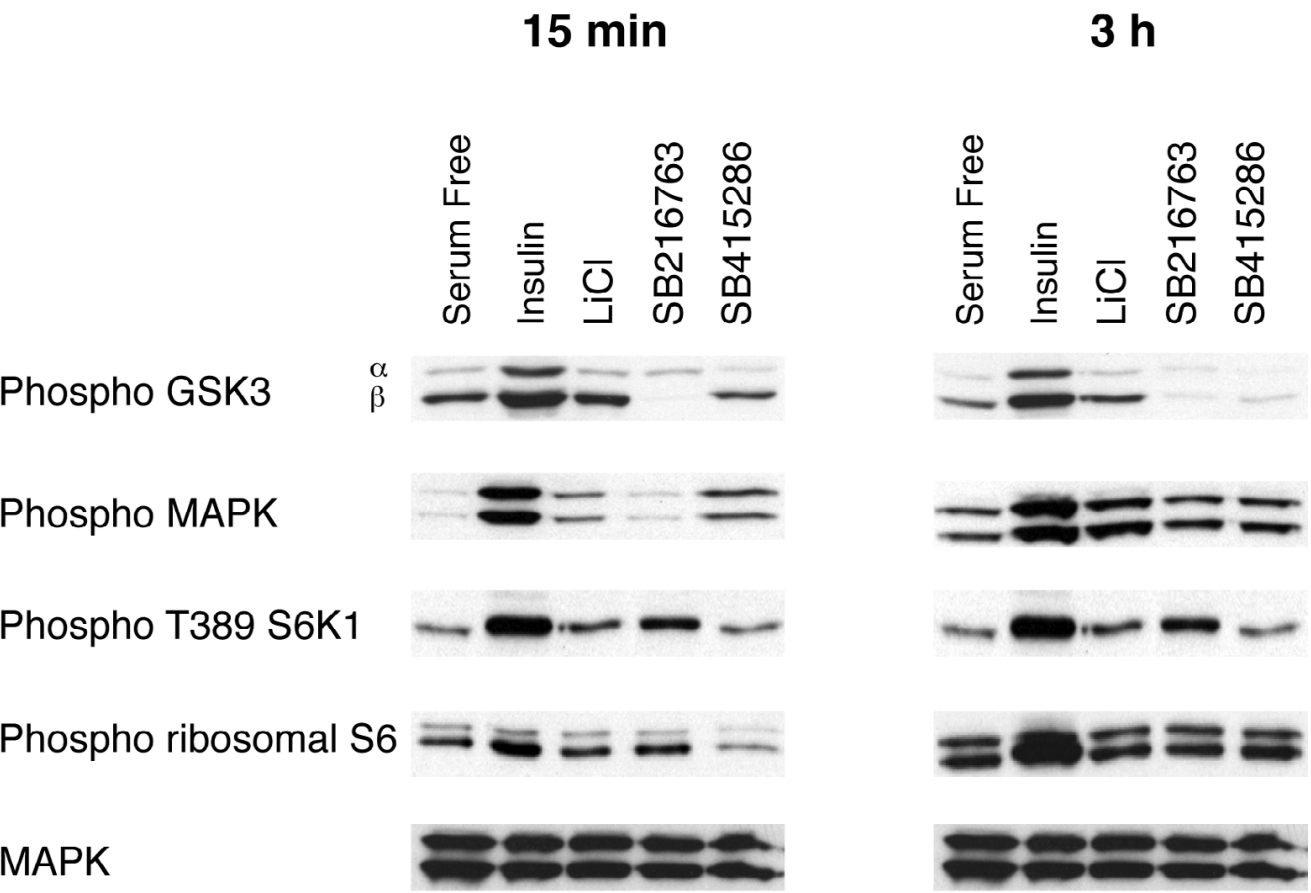
*Inhibition of GSK-3 does not affect the phosphorylation of MAPK or regulation of the mTOR pathway*. H4IIE cells were serum starved overnight prior to incubation with insulin, 10 nM; lithium chloride, 20 mM; SB216763, 30 μM; or SB415286, 100 μM for 15 min (A) or 3 h (B). Cells were lysed, and the lysates subjected to SDS PAGE as described in materials and methods, transferred to nitrocellulose and immunoblotted with antibodies as labelled (Phospho; phosphospecific antibody). Similar results were obtained from two experiments carried out in duplicate

SB 214763 and SB 415286 are cell-permeable maleimide compounds that selectively inhibit GSK-3 [29]. Treatment of H4IIE cells with either compound reduces IGFBP-1 gene expression (Fig 2). Expression is more sensitive to SB 214763 than SB 415286 (consistent with its lower IC-50 towards GSK-3 *in vitro *[29]). Importantly, cyclophilin mRNA levels remain unchanged in the presence of these compounds. Furthermore, under these conditions the regulatory phosphorylations of PKB and FOXO-1 are unaffected by SB214763, SB415286 or lithium [28]. In addition, SB214763 or SB415286 do not affect the phosphorylation of Ser-9 (GSK-3β) or Ser-21 (GSK-3α). Similarly, MAPK and S6K activity are not significantly affected by these compounds, as judged by the phosphorylation status of these insulin-regulated signalling molecules (Fig 3). Hence, the effects seen with these compounds on IGFBP-1 are likely to be due to the inhibition of GSK-3 rather than as a consequence of down/up-regulation of PKB, FOXO-1, MAPK or the mTOR pathway, which are known to effect IGFBP-1 gene expression.

### Paullones are potent inhibitors of GSK-3 that reduce IGFBP-1 gene expression

**Figure 4:**
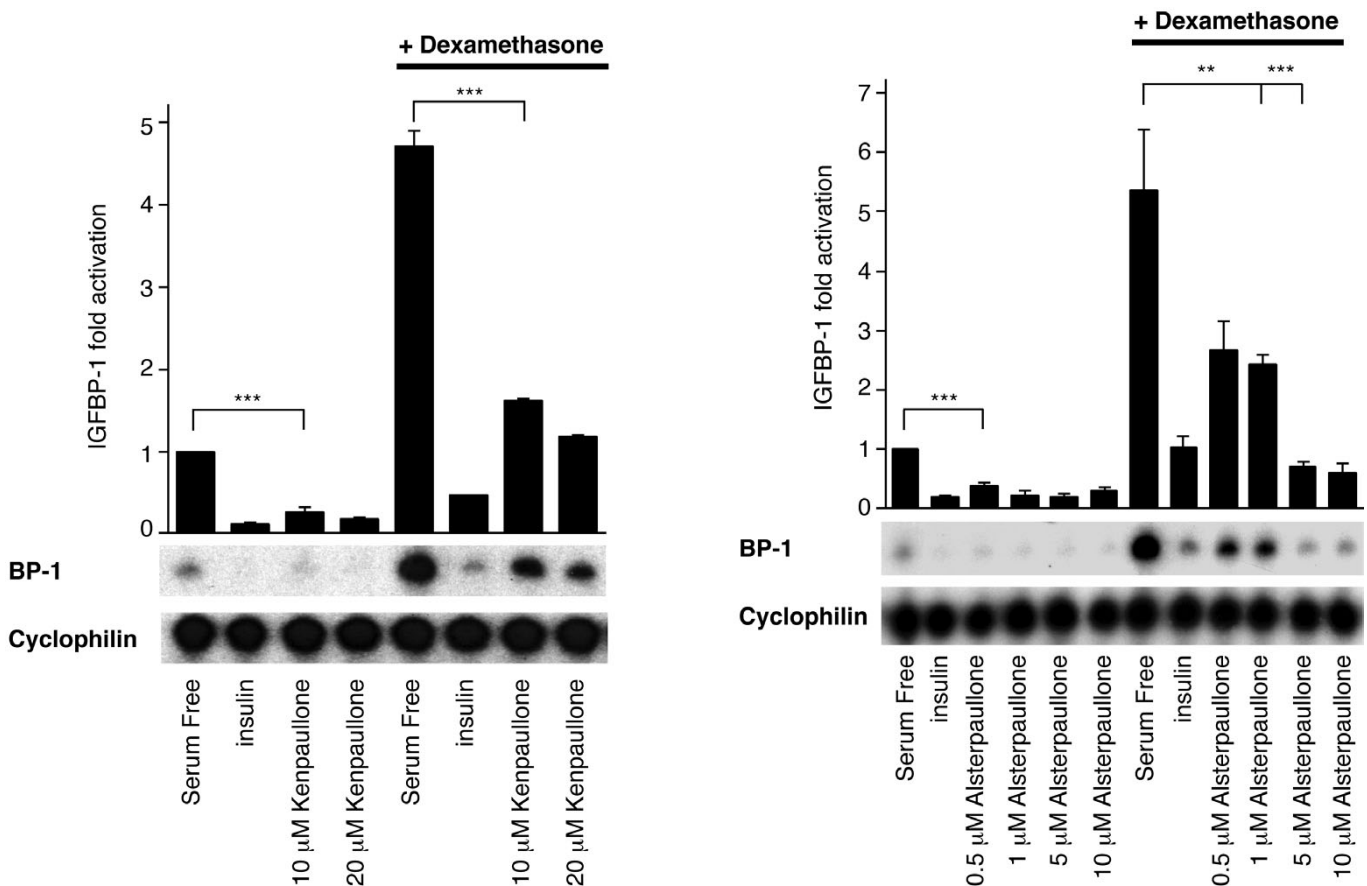
*Paullones reduce IGFBP-1 gene expression*. H4IIE cells were serum starved overnight prior to a 3 h incubation with insulin,10 nM; dexamethasone, 500 nM; plus or minus kenpaullone (A) or alsterpaullone (B) at the concentrations shown. Total cellular RNA was isolated and an RNase protection assay was performed, as described in material and methods. Results are presented as fold induction relative to control (serum free) and are means ± standard error of two experiments performed in duplicate (upper panels). Representative experiments are also shown (lower panels). ***, p < 0.001 and ** p < 0.01

**Figure 5:**
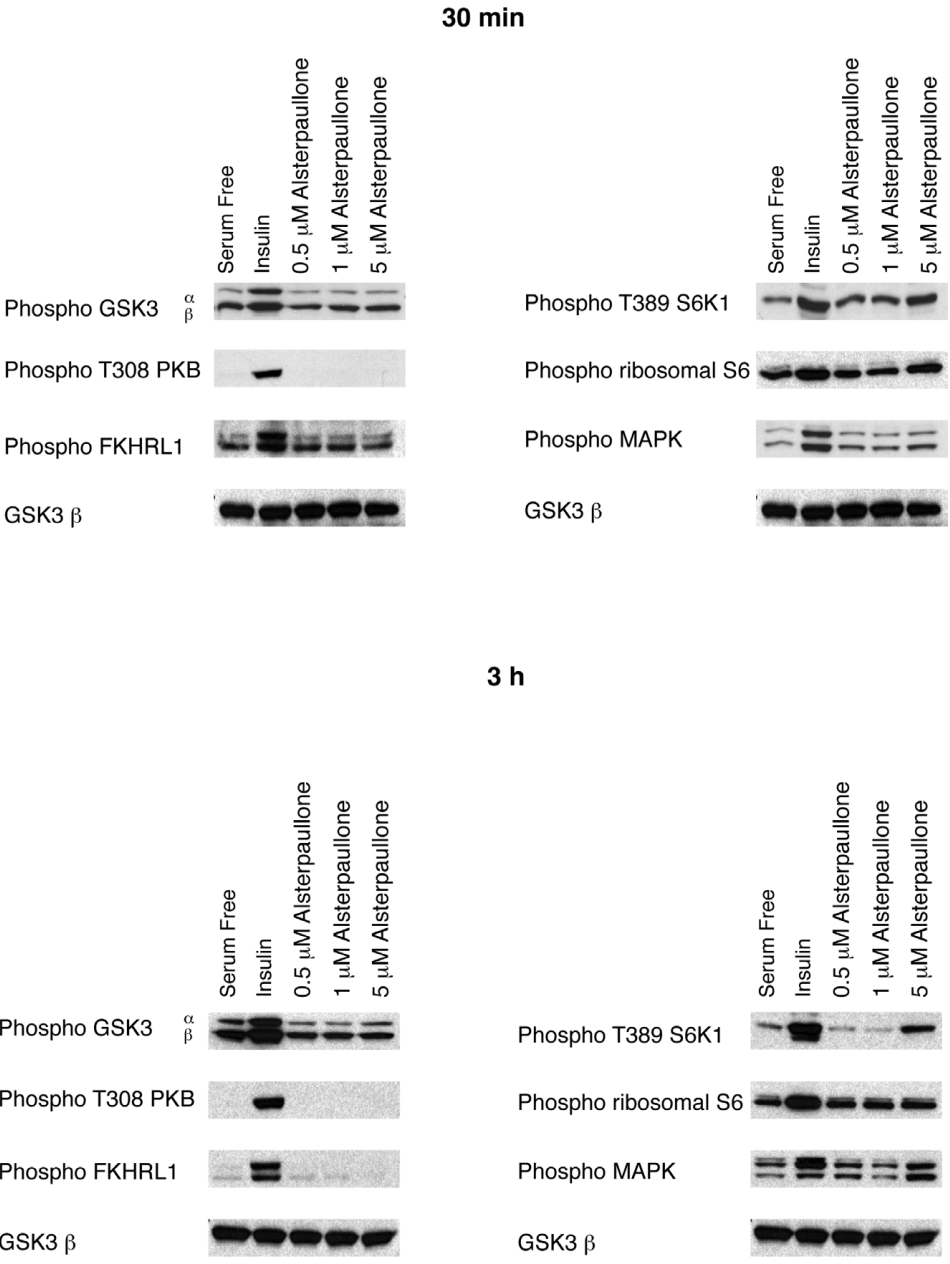
*Alsterpaullone does not affect the regulatory phosphorylation sites of PKB, FOXO-1, MAPK, and components of the mTOR pathway*. H4IIE cells were serum starved overnight prior to incubation with 10 nM insulin, or alsterpaullone at the concentrations shown for 30 min (A) or 3 h (B). Cells were lysed, and the lysates subjected to SDS PAGE, transferred to nitrocellulose and immunoblotted with antibodies as labelled (Phospho; phosphospecific antibody). Similar results were obtained from two experiments carried out in duplicate

Paullones are a family of benzazepinones that are potent (IC50; 20–200 nM), ATP-competitive inhibitors of cyclin-dependent kinases (CDKs) and the closely related neuronal CDK5/p25 [30-32]. Subsequently, they have been shown to be very potent inhibitors of GSK-3β [33]. Two members of this family, kenpaullone and alsterpaullone, reduce IGFBP-1 gene expression in a dose dependent manner (Fig 4). Alsterpaullone is much more potent than kenpaullone, reducing IGFBP-1 mRNA levels by 90% at 5 μM compared to a 50% reduction seen with 10 μM kenpaullone (Fig 4). Once more, this is consistent with the lower IC50 of alsterpaullone toward GSK-3 *in vitro *[33]. Alsterpaullone (like the maleimides) does not affect the phosphorylation of PKB, FOXO-1, MAPK, S6K or S6 (Fig 5). Similarly, phosphorylation at residues Ser-9 (GSK-3β) and Ser-21 (GSK-3α) of GSK-3 is unaffected by alsterpaullone treatment (Fig 5). Phosphorylation of Thr-308 (PKB) correlates with the activation of PKB while phosphorylation of Ser-9 (GSK-3β), Ser-21 (GSK-3α) and Thr-32 (FKHRL1) is indicative of inhibition of these PKB substrates.

### CHIR99021, the most specific GSK-3 inhibitor reported to date, also represses IGFBP-1 gene expression

**Figure 6:**
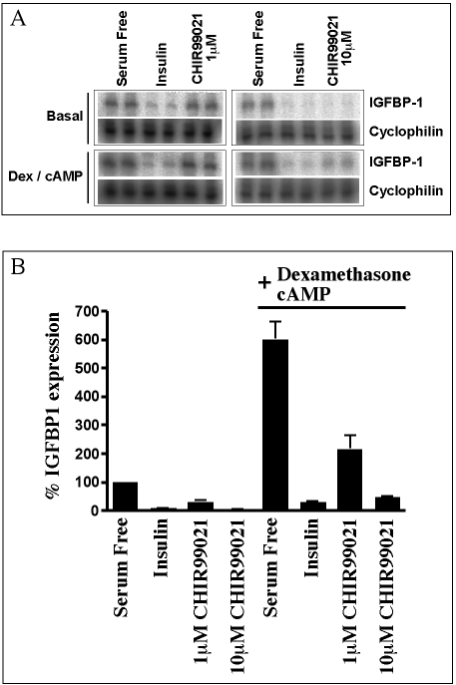
*CHIR99021 reduces IGFBP-1 gene expression*. H4IIE cells were serum starved overnight prior to a 3 h incubation with insulin,10 nM; dexamethasone, 500 nM; 8CPT-cAMP, 0.1 mM; plus or minus CHIR99021 at the concentrations shown. Total cellular RNA was isolated and an RNase protection assay was performed to measure IGFBP-1 and cyclophilin mRNA, as described in material and methods. Representative experiments are shown (A), while results are presented (B) as % expression (after correction for cyclophilin expression), relative to control (serum free) and are means ± standard error of two experiments performed in duplicate.

Although alsterpaullone, kenpaullone, SB214763, and SB415286 are potent inhibitors of GSK-3, they also exhibit activity against CDKs. However, the aminopyrimidine CHIR99021 shows 350-fold selectivity toward GSK-3 compared to CDKs (Jenny Bain and Sir Philip Cohen, University of Dundee, personal communication), and exhibits a Ki of < 10 nM in vitro [34]. It is the most selective inhibitor of this enzyme reported to date [34,35]. Treatment of H4IIE cells with CHIR99201 dramatically reduced basal and glucocorticoid-induced IGFBP-1 gene transcription, at concentrations between 1 and 10 μM (Fig 6)

### CHIR99021 reciprocally regulates β-catenin activity and IGFBP-1 gene transcription

**Figure 7:**
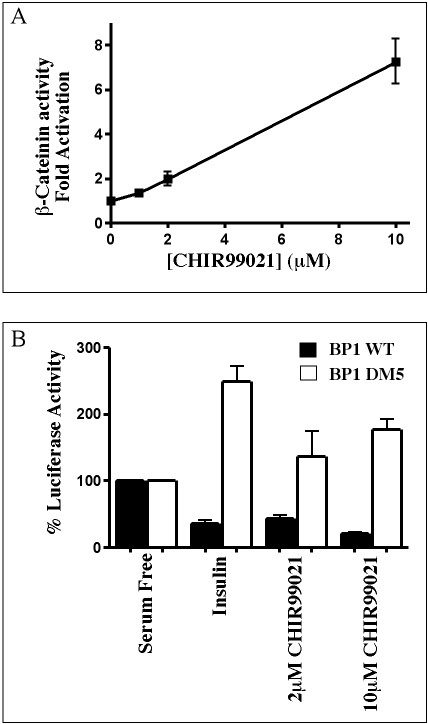
*CHIR99021 regulates both β-catenin activity and TIRE containing promoter activity*. H4IIE cells were transfected with TOPFlash (A) or alternatively with BP-1 WT or BP-1 DM5 (B) reporter constructs. Cells were incubated for 24 h with 10 nM insulin or CHIR99021 at the concentrations shown, prior to lysis and luciferase assays as described in materials and methods. Results are presented as fold induction relative to basal luciferase activity (no inhibitor) (A) or % luciferase activity relative to basal (serum free) luciferase expression (B) and are the means ± standard error of at least two experiments performed in triplicate. The basal activity of BP-1 WT and BP-1 DM5 is not significantly different.

H4IIE cells were transiently transfected with a luciferase-reporter construct containing TCF/LEF binding sites, whose activity is regulated by the GSK-3 substrate, β-catenin. Inhibition of GSK-3 results in the accumulation of β-catenin in the cytoplasm where it can form complexes with TCF/LEF. The complex translocates to the nucleus and activates transcription of target genes. Treatment of transfected H4IIE cells with CHIR99021 results in a dose-dependent increase in luciferase activity, regulated by the β-catenin/TCF complex (Fig 7A). The β-catenin mediated transcription is induced two-fold by 2 μM CHIR99021, reaching six to seven-fold at 10 μM. Therefore the concentration required to induce β-catenin activity is equivalent to that required for reduction of endogenous IGFBP-1 mRNA (Fig 6).

Meanwhile, insulin treatment of H4IIE cells previously transfected with a luciferase reporter construct under the control of a thymidine kinase promoter containing the IGFBP-1 TIRE (BP-1WT), reduces luciferase expression by 60% (Fig 7B). This effect is abolished by a two base pair mutation of the TIRE (BP-1DM5) (Fig 7B and [36]). Interestingly, 2 μM CHIR99021 reduces BP-1 WT activity by around 50% (Fig 7B), while 10 μM inhibits luciferase expression by 70%, with no effect on BP-1 DM5 activity. This demonstrates that CHIR99021 reduces TIRE activity, at a concentration that also induces β-catenin-mediated gene transcription (2–10 μM). This strongly argues that the effects of CHIR99021 on TIRE activity are mediated through inhibition of GSK-3.

### Enhanced expression of GSK-3 reduces insulin regulation of the IGFBP-1 TIRE

**Figure 8:**
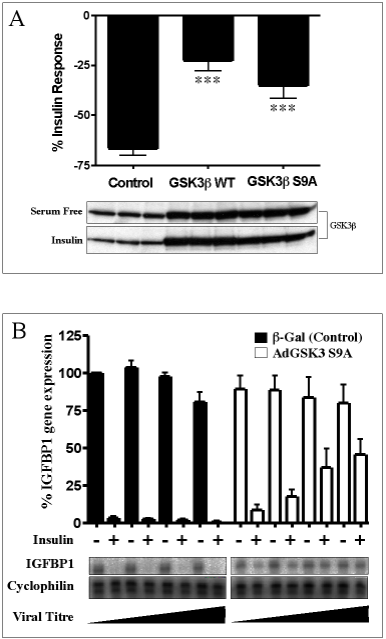
*Overexpression of GSK-3β reduces insulin regulation of the IGFBP-1 TIRE*. H4IIE cells were infected with adenovirus expressing either β-Galactosidase (control), GSK-3β (GSK-3wt), or insulin-insensitive GSK-3β (GSK-3S9A). A) Cells were incubated for 16 hours before transfection with 10 μg of BP-1 WT as described under the Methods section. After 24 hours at 37°C +/- insulin (10 nM) cells were lysed and either luciferase assays performed (upper panel) or GSK-3 levels determined by Western Blot (lower panel). Results in the upper panel are presented as % insulin repression of luciferase expression and are the means +/- S.E.M. of at least five experiments performed in duplicate or triplicate. Basal luciferase expression is 3-fold higher in WT and S9A-GSK-3 infected cells compared with control. The lower panels provide a representative analysis of expression of GSK-3 (in triplicate) in each treatment. There was a significant reduction in the effect of insulin on BP-1 WT when either GSK-3 WT (***p < 0.001, control vs WT) or GSK-3 S9A (***p < 0.001, control vs S9A) was overexpressed. There is no significant difference between the WT and S9A data sets (p = 0.160). B) After infection cells were incubated for 24 hr prior to a 3 hr incubation with hormones as indicated. Cells were lysed and IGFBP-1 and cyclophilin mRNA levels assessed by RNase Protection Assay. A representative experiment is shown (lower panel), while relative mRNA levels ± SEM are presented for two experiments performed in duplicate in the upper panel.

In order to assess the requirement for inhibition of GSK-3 in insulin regulation of the IGFBP-1 TIRE we over expressed wild-type GSK-3 (GSK-3β-WT), insulin-insensitive GSK-3 (GSK-3β-S9A) or control protein (β-galactosidase) in H4IIE cells using adenoviral vectors. Infected cells were subsequently transfected with BP-1-WT and treated with or without insulin (Fig 8A). The inhibitory effect of insulin on the BP-1 TIRE was significantly reduced when GSK-3 was over expressed (Fig 8A), demonstrating that inhibition of GSK-3 is required for full repression of this element by insulin. Both wild-type (p < 0.001) and S9A-GSK-3 (p < 0.001) over expression (around 3 to 5-fold increase in expression) reduced insulin regulation of this element. Meanwhile, adenoviral expression of GSK-3β-S9A also reduced the ability of insulin to repress IGFBP-1 mRNA in the H4IIE cells (Fig 8B).

### CHIR99021 does not regulate FOXO-1 transactivation potential

**Figure 9:**
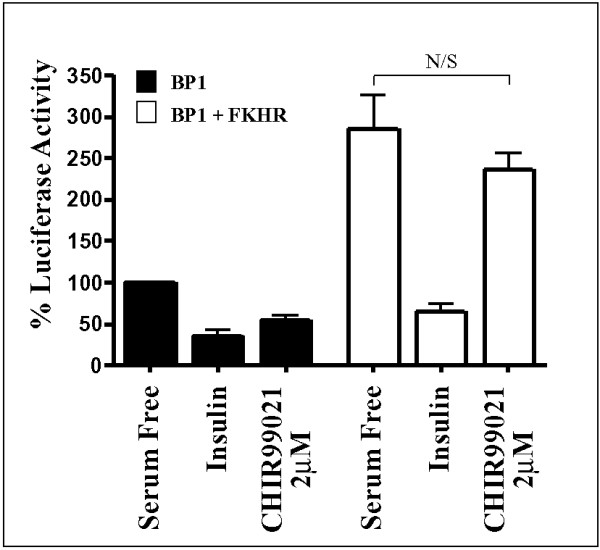
*CHIR99021 does not affect FOXO-1 transactivation potential*. H4IIE cells were transfected with BP-1WT along with pEBG2T or pEBG2T-FOXO-1. Cells were incubated with 10 nM insulin or 2 μM CHIR99021 for 24 h prior to lysis and luciferase assays as described in materials and methods. Results are presented as % luciferase activity relative to basal (serum free) luciferase expression and are the means ± S.E. of two experiments performed in triplicate. NS; not significant.

BP-1 TIRE activity can be regulated by co-expression of FOXO-1 [22,36]. Therefore, we examined the effect of CHIR99021 on the ability of FOXO-1 to regulate TIRE activity. When FOXO-1 is co-expressed with BP-1 WT in H4IIE cells, the expression of luciferase is induced around 3-fold (Fig 9). Insulin inhibits this FOXO-1-induced luciferase activity, while sensitivity to 2 μM CHIR99021 is completely lost in the presence of co-expressed FOXO-1 (Fig 9). The concentration of FOXO-1 used is less than maximal for induction of the BP-1 WT. This data suggests that FOXO-1 overexpression desensitises the TIRE to CHIR99021 and therefore that GSK-3 does not significantly regulate FOXO-1 activity.

## Discussion

### GSK-3 activity is required for IGFBP-1 promoter activity through direct regulation of the TIRE

This study demonstrates that six agents, of four different chemical classes, which share an ability to inhibit GSK-3 mimic the effect of insulin on IGFBP-1 gene expression. This is reminiscent of the effect of lithium ions, SB216763 and SB415286 on two other insulin repressed gene promoters, PEPCK and G6Pase [28]. Indeed, a heterologous promoter containing the IGFBP-1 TIRE (a related sequence is common to all three of these insulin-regulated gene promoters), is also inhibited by CHIR99021 (Fig 7B). Similar promoter sequences are important for the insulin regulation of the tyrosine aminotransferase [37], aspartate aminotransferase [38], IRS-2 [39], and HMG CoA Synthase [40] gene promoters. Our data would predict that all of these genes, and any other promoters containing a TIRE, are likely to be repressed by treatment of cells with inhibitors of GSK-3. This provides an apparent paradox since we and others have found that insulin does not regulate every TIRE-containing gene promoter by an identical mechanism. For example, insulin regulation of the IGFBP-1 (but not the PEPCK or G6Pase) gene promoter requires mTOR activity [10,25,26]. Meanwhile, FOXO-1 is a TIRE-binding protein that has been proposed to regulate these three genes. However, cells that stably overexpress FOXO-1 show increased G6Pase but not PEPCK expression [41], and genetic manipulation of FOXO-1 has differential effects on these three gene promoters [19]. These data demonstrate that distinct signalling mechanisms control the regulation of these three TIRE-containing genes. Therefore, each TIRE structure may require GSK-3 activity for function but distinct signalling networks link each gene promoter with the insulin receptor. The common requirement for GSK-3 activity suggests that a GSK-3 substrate is key for the initiation of gene transcription for each TIRE-containing promoter.

### Inhibition of GSK-3 is required for full inhibition of the IGFBP-1 TIRE by insulin

Insulin induces PKB activity, promoting phosphorylation of Ser-21 of GSK-3α and Ser-9 of GSK-3β, thereby reducing total GSK-3 activity by between 20 and 80%, dependent on cell type. Therefore, expression of a mutant GSK-3β with Ser-9 replaced by alanine renders cellular GSK-3 activity insensitive to insulin [14]. Indeed, expression of this mutant significantly reduces the ability of insulin to repress BP-1 WT (Fig 8A), or the endogenous gene promoter (Fig 8B), demonstrating that insulin requires to inhibit GSK-3 for full repression of this gene promoter element. Similarly, four to five fold over expression of wild-type GSK-3β antagonises insulin repression of the BP-1 WT (Fig 8A). Although insulin will promote phosphorylation and inhibition (50–60% in H4IIE cells) of this recombinant GSK-3 in cells, the overall activity remains higher than un-stimulated control cells. This suggests that insulin must reduce GSK-3 activity below a threshold in order to fully repress BP-1 WT. This is the first demonstration of an absolute requirement for GSK-3 inhibition in insulin regulation of gene transcription.

### What is the molecular link between GSK-3 and the TIRE?

The GSK-3 inhibitors regulate IGFBP-1 gene expression in the absence of regulation of PKB, MAP kinase, FOXO-1 or mTOR ([28] and Figs 3 and 4), known regulators of the IGFBP-1 promoter. This suggests a more direct regulation of this element, possibly of a TIRE-interacting protein itself. There are numerous transcription factors that have been proposed to be substrates for GSK-3 *in vitro *and in some cases *in vivo *(for review see [42]). These include β-catenin, c-jun, CREB, glucocorticoid receptor (GR) and c-myc. The phosphorylation of β-catenin [43,44], c-jun [45,46], GR [47] and c-myc [48] by GSK-3 promotes their destruction or reduces their activity, while the phosphorylation of CREB (at Ser-129) is thought to increase CREB activity [49], although this has been subsequently questioned [50]. Since inhibition of GSK-3 reduces TIRE activity, one presumes that GSK-3 mediated phosphorylation of a TIRE-binding protein would result in its activation (although possibly a permissive effect allowing activation by an additional mechanism), nuclear localisation or stabilisation. This would seem to rule out β-catenin, c-jun, GR and c-myc in the GSK-3-mediated regulation of the TIRE. Meanwhile CREB does not bind directly to a TIRE *in vitro*. The only known GSK-3 substrates that have been demonstrated to bind to or regulate the TIRE are members of the CAAT-enhancer binding protein (C/EBP) family of transcription factors. GSK-3 phosphorylates C/EBPα at Thr-222/Thr-226 [51] while C/EBPβ can regulate TIRE activity and is itself regulated by insulin [52]. The reported regulation of C/EBPβ by insulin is PI 3-kinase and PKB-dependent but is mediated through phosphorylation of the co-regulator protein p300/CBP [52]. We are currently examining whether the GSK-3 inhibitors regulate C/EBP and p300 phosphorylation and/or activity. Meanwhile, Granner and colleagues have found that insulin treatment of H4IIE cells increases the cellular levels of LIP (an inhibitory form of C/EBPβ that lacks the p300/CBP binding and activation domain) [53]. LIP subsequently replaces LAP (the activating form of C/EBPβ) on the endogenous PEPCK promoter. This prevents the recruitment of RNA polymerase II and p300/CBP, eventually leading to the repression of PEPCK gene expression. However, the LIP/LAP interacting elements within the PEPCK promoter are distinct from the TIRE [53]. Finally, our data suggests that the effect of GSK-3 inhibitors is independent of regulation of FOXO-1 (Figs 4 and 9), the best characterised TIRE-binding protein. Therefore, much more work will be required to identify the GSK-3 substrate that regulates this DNA element.

### GSK-3 inhibitors as therapeutics

Agents that mimic the physiological processes that are regulated by insulin have the potential to be of therapeutic value for the treatment of insulin resistant states such as diabetes. Lithium chloride, SB216763, SB415286 and CHIR99021 inhibit GSK-3 and therefore mimic many of the actions of insulin. For instance, lithium chloride stimulates glucose transport and glycogen synthesis in adipocyte and muscle cell lines [54-56], while SB216763 and SB415286 stimulate glycogen synthesis in hepatocytes [29]. Meanwhile, CHIR99021 potentiates insulin activation of glucose transport and utilisation in vitro and in vivo [34], and related compounds reduce muscle insulin resistance [57] in animal models of diabetes. We have found that GSK-3 inhibitors also mimic the ability of insulin to repress key metabolic genes such as PEPCK, G6Pase and IGFBP-1 ([28] and Figs 1, 2 and 4). Studies in animal models of diabetes suggest that these agents alleviate hyperglycaemia through both activation of glycogen synthesis and inhibition of hepatic glucose production [58,59]. However, a vast number of biological processes are known to be regulated by GSK-3, thereby questioning their long term use as regulators of glucose homeostasis. Importantly, GSK-3 associates with and regulates proteins linked to the development of colonic cancer (APC, axin and β-catenin). Meanwhile, ablation of one of the two genes for GSK-3 (GSK-3β) in mice proved to be fatal due to increased hepatic sensitivity to TNFα-induced apoptosis [60]. Despite all of the potential problems that may be associated with GSK-3 inhibitors, deleterious effects of such compounds in animals remain to be formally reported. Currently, GSK-3 inhibitors are being investigated for the treatment of numerous psychiatric disorders [61,62], neurodegeneration [63,64] and even hair loss [65].

## Conclusions

The work presented herein demonstrates for the first time that inhibition of GSK-3 is required for complete insulin regulation of IGFBP-1, while we have identified the DNA element by which GSK3 targets this gene promoter. As such, GSK-3 inhibition will mimic the insulin regulation of IGF1 bio-availability, as well as reducing the expression of hepatic gluconeogenic genes. It remains to be seen how many other insulin-regulated (and/or TIRE-containing) gene promoters are sensitive to these inhibitors.

## Methods

### Materials

Radioisotopes were obtained from Amersham, Bucks, UK ({γ^32^P}-ATP) and ICN, Thame, Oxfordshire, UK ({α^32^P}-UTP). Insulin was purchased from Novo Nordisk, (Crawley, West Sussex, UK), kenpaullone and alsterpaullone from Calbiochem (La Jolla, CA) and the RNase Protection Assay Kit II from AMS Biotech/Ambion, (Austin, Texas). All other chemicals were of the highest grade available.

### Synthesis of CHIR 99021

CHIR99021 (6-{2-[4-(2,4-Dichloro-phenyl)-5-(4-methyl-1*H*-imidazol-2-yl)-pyrimidin-2-ylamino]-ethylamino}-nicotinonitrile) was synthesized in 7% overall yield using a convergent approach from 2,4-dichlorobenzoyl chloride and 6-chloro nicotinonitrile respectively ([66] and refs within).

### Cell Culture

The rat hepatoma cell line H4IIE was maintained in Dulbecco's Modified Eagle's medium (DMEM) containing 1000 mg/l glucose, 5% (v/v) foetal calf serum, as described previously [67]. Cells were incubated with hormones, at 37°C, for the times and at the concentrations indicated in the figure legends.

### RNA isolation and RNase protection assay

H4IIE cells were serum-starved overnight and treated with hormone/inhibitor for the times and at the concentrations indicated in the figure legends. Total cellular RNA was isolated using TriReagent™ (Sigma) following the manufacturer's instructions. An RNase Protection Assay (RPA) was performed to determine the relative amounts of IGFBP-1 and cyclophilin mRNA in each sample [26]. Band intensity was quantified on a phosphorimager (Fuji), data calculated as a ratio of IGFBP-1 to cyclophilin mRNA and presented as fold activation (for induced samples) where the intensity of control samples were set at one, or as % gene expression (for non-induced samples) where the level of gene expression in untreated cells is set at 100%.

### Preparation of cell extract for western blot

H4IIE cells were incubated in serum-free medium with hormones and inhibitors for the times and at the concentrations indicated in the figure legends. Cells were then scraped into ice-cold lysis buffer (25 mM Tris/HCl, pH 7.4, 50 mM NaF, 100 mM NaCl, 1 mM sodium vanadate, 5 mM EGTA, 1 mM EDTA, 1% (v/v) Triton X-100, 10 mM sodium pyrophosphate, 1 mM benzamidine, 0.1 mM PMSF, 0.27 M sucrose, 2 μM microcystin and 0.1% (v/v) 2-mercaptoethanol). Cell debris was removed by centrifugation at 13000 × g for 5 min and the protein concentration determined by the method of Bradford, using BSA as an internal standard.

### Antibodies for western blot analysis

Antibodies to phospho ribosomal protein S6 (Ser-235), phospho-FKHR-L1 (Thr-32) and GSK-3β were purchased from Upstate (Lake Placid, USA), while the phospho-specific Ser9/Ser21 GSK-3, Thr-308 PKB, Thr389-S6K1, and Thr-183/Tyr185 p42/p44 MAPK antibodies were purchased from Cell Signalling Technologies (Hertfordshire, UK). H4IIE cell lysates were prepared following incubation with hormones as described in figure legends and analysed by Western blot analysis.

### Plasmids

The plasmids BP-1 WT and BP-1 DM5 were a gift from Dr Robert Hall and Professor Daryl K. Granner (Vanderbilt University, TN, USA) [36]. The BP-1 WT plasmid represents a luciferase reporter construct under the control of a thymidine kinase promoter containing the IGFBP-1 TIRE wild-type sequence (5'-CAAAACAAACTTATTTTG). Two base pair mutations of the wild-type TIRE sequence at residues equivalent to position 5 of each A and B site (5'-CAAAAGAAACTTCTTTTG) produces a mutant promoter (BP-1 DM5) that is no longer responsive to insulin [36]. The FOXO-1 constructs have been described previously (10).

### Transient transfections

The TOPflash reporter plasmid kit were obtained from Upstate Biotechnology (Lake Placid, USA). TOPflash has Tcf binding sites driving luciferase expression. Tranfections were performed using the calcium phosphate procedure as described previously [10]. H4IIE cells were transfected in 10 cm dishes with BP-1 WT (10 μg), BP-1 M5 (10 μg), TOPFlash (10 μg), plus or minus 2 μg of GST-FOXO-1 as indicated. Cells were then incubated for 24 h in serum free media with or without hormones or inhibitors as described in figure legends. Cells were lysed in 300 μl lysis buffer (Promega, UK), the cell debris removed by centrifugation at 13000 × g for 2 min and the supernatant stored at -70°C. Luciferase assays were performed using the firefly luciferase assay system (Promega, UK), as per manufacturer's instructions, with luciferase activity being corrected for the protein concentration in the cell lysate.

### Adenoviral infection

H4IIE cells were infected with virus between a titre of 10^8 ^and 10^9 ^plaque forming units per ml, incubated at 37°C for 16 hr. Cells were then transfected with 10 μg of BP-1 WT as described above and incubated for a further 24 hr in the presence or absence of 10 nM insulin. Luciferase was harvested and assayed or cell extracts were prepared for western blot analysis, as described earlier.

### Statistical analyses

As a measure of statistical significance of differences in experimental groups, student t-tests were performed and 5% confidence limits applied.

## Abbreviations

G6Pase, glucose 6-phosphatase; IGFBP-1, IGF-binding protein-1; phosphatidyl inositol 3, kinase, PI 3-kinase; TIRE, thymine rich insulin response element; PKB, protein kinase B; PEPCK phosphoenolpyruvate carboxykinase; GSK-3, Glycogen synthase kinase 3

## Authors contributions

The majority of the data was obtained in equal measure by D.F. and S.P, the CHIR99021 was synthesised, purified and analysed by N.S. and R.M., the adenoviral vectors were produced and characterised by L.M.D. and C.J.R., while the project was conceived and supervised by C.S.
